# Continuous Manufacturing of Recombinant Drugs: Comprehensive Analysis of Cost Reduction Strategies, Regulatory Pathways, and Global Implementation

**DOI:** 10.3390/ph18081157

**Published:** 2025-08-04

**Authors:** Sarfaraz K. Niazi

**Affiliations:** College of Pharmacy, University of Illinois, Chicago, IL 60612, USA; sniazi3@uic.edu; Tel.: +1-312-297-0000

**Keywords:** continuous manufacturing, biosimilars, recombinant proteins, process analytical technology, perfusion bioreactors, ICH Q13, regulatory compliance, monoclonal antibodies, downstream processing, bioprocess optimization

## Abstract

The biopharmaceutical industry is undergoing a fundamental transformation from traditional batch manufacturing to continuous manufacturing (CM) for recombinant drugs and biosimilars, driven by regulatory support through the International Council for Harmonization (ICH) Q13 guidance and compelling economic advantages. This comprehensive review examines the technical, economic, and regulatory aspects of implementing continuous manufacturing specifically for recombinant protein production and biosimilar development, synthesizing validated data from peer-reviewed research, regulatory sources, and global implementation case studies. The analysis demonstrates that continuous manufacturing offers substantial benefits, including a reduced equipment footprint of up to 70%, a 3- to 5-fold increase in volumetric productivity, enhanced product quality consistency, and facility cost reductions of 30–50% compared to traditional batch processes. Leading biomanufacturers across North America, Europe, and the Asia–Pacific region are successfully integrating perfusion upstream processes with connected downstream bioprocesses, enabling the fully end-to-end continuous manufacture of biopharmaceuticals with demonstrated commercial viability. The regulatory framework has been comprehensively established through ICH Q13 guidance and region-specific implementations across the FDA, EMA, PMDA, and emerging market authorities. This review provides a critical analysis of advanced technologies, including single-use perfusion bioreactors, continuous chromatography systems, real-time process analytical technology, and Industry 4.0 integration strategies. The economic modeling presents favorable return-on-investment profiles, accompanied by a detailed analysis of global market dynamics, regional implementation patterns, and supply chain integration opportunities.

## 1. Introduction and Historical Context

The pharmaceutical manufacturing industry has remained fundamentally anchored to batch processing methodologies since it emerged from traditional apothecary practices in the mid-19th century [1]. Major pharmaceutical enterprises, including Merck (Darmstadt, Germany, established in 1668), Pfizer (Brooklyn, NY, USA, 1849), Eli Lilly (Indianapolis, IN, USA, 1876), and Bayer (Barmen, Germany, 1863), all evolved from small-scale apothecary operations into industrial batch manufacturing operations during the Industrial Revolution [2]. This historical commitment to batch processing has persisted despite the successful implementation of continuous manufacturing in adjacent industries, most notably the chemical and petrochemical sectors, where continuous processing has been the standard for over a century [3] (Table 1).

The chemical industry established the first continuous manufacturing process for sulfuric acid production at the beginning of the 19th century, demonstrating the fundamental principles of continuous flow chemistry that would later influence modern pharmaceutical applications [4]. The petrochemical industry further advanced continuous processing when Union Carbide constructed the world’s first petrochemical plant in West Virginia in 1920, utilizing continuous separation and thermal cracking techniques to convert ethane into ethylene with unprecedented efficiency and consistency [5].

The pharmaceutical industry’s historical reluctance to adopt continuous manufacturing stems from several fundamental differences compared to traditional chemical processes [6]. Pharmaceutical manufacturing, particularly biomanufacturing, involves complex biological systems that introduce inherent variability and require sophisticated control strategies [7]. Additionally, the regulatory environment for pharmaceuticals has traditionally favored well-understood batch processes with established quality control paradigms based on lot release testing rather than real-time process control [8].

However, the economic pressures facing the pharmaceutical industry, particularly in the biopharmaceutical sector, have created compelling drivers for technological innovation [9]. The average cost of developing a biotechnology drug reached approximately USD 1.9 billion as of 2012, with subsequent estimates suggesting even higher development costs due to increased regulatory requirements and longer development timelines [10]. Simultaneously, healthcare systems worldwide are experiencing unprecedented cost pressures, with biologics representing an increasingly significant portion of pharmaceutical expenditures, reaching USD 487 billion in the United States alone in 2024 [11].

The convergence of these economic pressures with advances in process technology, analytical methods, and regulatory science has laid the groundwork for the current transition toward continuous manufacturing in biopharmaceutical production [12]. The recognition that continuous processing could potentially achieve significant cost savings while maintaining or improving product quality has generated substantial industry interest and regulatory support, culminating in the development of comprehensive regulatory guidance through the ICH Q13 framework [13,14].

## 2. Comprehensive Analysis of ICH Q13 Regulatory Framework and Global Implementation

**Figure 1:**
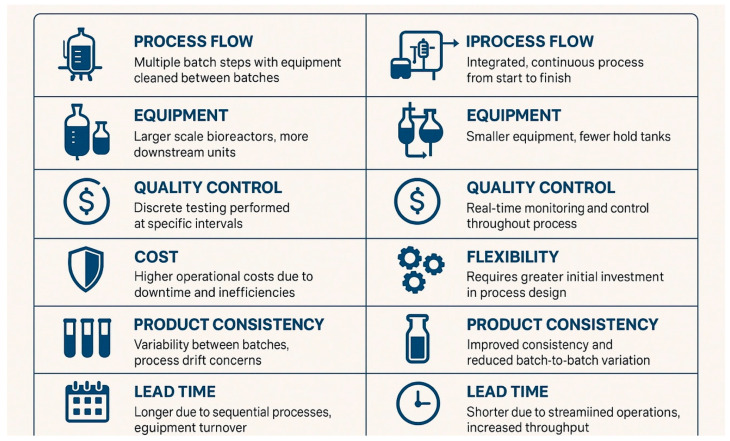
Comparison diagram illustrating key differences between batch and continuous manufacturing process flows.

The International Council for Harmonization (ICH) Q13 guidance marks a watershed moment in pharmaceutical regulatory science, providing the first comprehensive, globally harmonized framework for implementing continuous manufacturing [13]. The guidance development process spanned multiple years of international collaboration between regulatory agencies, industry stakeholders, and academic institutions, reflecting the complexity and significance of transitioning from traditional batch paradigms to continuous manufacturing approaches [15] (Figure 1, Table 2).

### 2.1. ICH Q13 Structure and Scope

The ICH Q13 guidance comprises a comprehensive 39-page document structured as a primary document supplemented by five detailed annexes that address specific implementation scenarios [13]. The main guidance document provides 15 pages of fundamental principles covering development approaches, implementation strategies, operational considerations, and lifecycle management requirements [17]. The accompanying annexes offer 24 pages of detailed, application-specific guidance addressing distinct manufacturing scenarios ranging from small molecule continuous manufacturing to complex therapeutic protein production systems [18].

The guidance establishes a clear definitional framework for continuous manufacturing, describing it as processes involving the continuous feed of input materials into the transformation of in-process materials within, and the concomitant removal of output materials from a manufacturing process [13]. This definition encompasses both fully integrated continuous systems, where all unit operations are connected in a continuous flow, and hybrid systems that strategically combine continuous and batch operations to optimize specific manufacturing objectives [19].

Annex III of ICH Q13 specifically addresses therapeutic protein drug substances, providing detailed guidance for the continuous manufacturing of recombinant proteins, monoclonal antibodies, and other biological products [16]. This annex acknowledges the unique challenges inherent to biological manufacturing systems, including the inherent variability of living cell systems, the complexity of downstream purification processes, and the critical importance of maintaining product quality and safety throughout continuous operation [20].

### 2.2. Quality by Design Framework Integration

The implementation of continuous manufacturing under ICH Q13 requires pharmaceutical companies to demonstrate a fundamentally enhanced level of process understanding compared to traditional batch manufacturing approaches, fully integrating Quality by Design principles [13]. The enhanced process understanding requirements encompass the detailed characterization of all critical process parameters and their relationships to critical quality attributes throughout the entire manufacturing process [21]. This characterization must extend beyond traditional batch process understanding to include dynamic process behavior, transient conditions during startup and shutdown, and the propagation of process disturbances throughout integrated continuous systems [22].

Control strategy development represents a fundamental departure from traditional batch manufacturing approaches, requiring real-time monitoring and control capabilities rather than relying primarily on end-product testing [23]. The control strategy must demonstrate the ability to detect, respond to, and correct process deviations in real time while maintaining product quality within predetermined specifications [24]. This requires the implementation of sophisticated process analytical technology, advanced process control systems, and comprehensive material diversion strategies for managing out-of-specification materials [25].

### 2.3. Regional Regulatory Implementation and Harmonization

Table 3 presents a comparative analysis of regulatory adaptations related to continuous manufacturing proposals (Table 3).

The global implementation of ICH Q13 has proceeded according to a carefully coordinated timeline designed to ensure harmonized adoption across major regulatory jurisdictions [13]. The United States Food and Drug Administration adopted the ICH Q13 guidance in March 2023, replacing previous draft guidance documents and establishing a unified regulatory framework for continuous manufacturing applications [26]. The European Medicines Agency implemented the guidance effective July 2023, establishing an Implementation Working Group to develop comprehensive training materials and provide ongoing support for manufacturers and regulatory reviewers [26].

Regulatory harmonization extends beyond the adoption of simple guidance to include coordinated training programs, shared review standards, and collaborative inspection approaches [31]. The FDA has established specialized review teams with enhanced expertise in continuous manufacturing technologies. At the same time, the EMA has developed specific training modules for regulatory assessors focusing on the unique aspects of continuous manufacturing evaluation [32].

Japan’s PMDA has developed specific technical guidance for continuous manufacturing, emphasizing process robustness and quality consistency, with a particular focus on biopharmaceutical applications [28]. China’s NMPA has established pilot programs for continuous manufacturing evaluation, offering expedited review pathways for innovative manufacturing technologies that demonstrate clear patient benefits [29]. Brazil’s ANVISA has implemented specialized pathways for biosimilar continuous manufacturing, recognizing the potential for cost reduction and improved healthcare access [30]. The Pan American Network for Drug Regulatory Harmonization is developing harmonized approaches for continuous manufacturing evaluation across Latin American markets [33].

## 3. Global Market Dynamics and Regional Implementation Patterns

### 3.1. Market Size and Economic Drivers

The global biopharmaceutical market has experienced unprecedented growth, with the worldwide biologic market projected to reach USD 444.40 billion in 2024, reflecting the increasing importance of biological therapeutics in modern healthcare [18,34]. This growth trajectory has been accompanied by escalating concerns about healthcare affordability, particularly regarding biological medications that often carry premium pricing due to complex manufacturing requirements and limited competition from biosimilar alternatives [35] (Table 4).

The economic case for continuous manufacturing in biopharmaceutical production is compelling when analyzed across multiple cost categories [38]. Traditional batch manufacturing of recombinant proteins requires substantial facility investments, with typical commercial-scale facilities requiring capital expenditures of USD 500 million to USD 2 billion, depending on capacity and product complexity [36]. These facilities are characterized by large-scale equipment, including bioreactors ranging from 15,000 to 25,000 L, extensive tank farms for intermediate storage, and multiple dedicated production suites for different products or production campaigns [39].

### 3.2. Biosimilar Market Opportunity and Impact

The biosimilar market represents a particularly compelling application for continuous manufacturing technologies due to the inherent cost-competitiveness requirements of biosimilar products [40]. Biosimilars typically require 20–30% price reductions compared to reference products to achieve meaningful market penetration, creating substantial pressure for manufacturing cost optimization [41] (Table 5).

Recent market analysis demonstrates the significant impact that cost-competitive biosimilars can achieve when manufacturing efficiencies enable aggressive pricing strategies [46]. The first biosimilar approved in the United States, filgrastim-sndz, achieved a combined 40% market share by volume within two years of its launch, with pre-rebate prices 30–45% lower than those of the reference biologic [47]. More recent biosimilar launches have demonstrated even greater success, with biosimilars of bevacizumab, trastuzumab, and rituximab achieving market shares of 82%, 80%, and 67%, respectively, within three years of their launch [48].

### 3.3. Regional Implementation Patterns and Success Stories

Table 6 provides a comparison of the implementation of continuous manufacturing across the globe (Table 6).

Genentech’s South San Francisco facility represents one of the most successful large-scale implementations of continuous manufacturing for monoclonal antibodies, achieving a 35% reduction in manufacturing costs while maintaining equivalent product quality [49]. The facility integrates perfusion cell culture with continuous downstream processing, enabling production flexibility across multiple product lines. Biogen’s Denmark facility has successfully implemented continuous manufacturing for various sclerosis therapeutics, achieving significant cost reductions while meeting the EMA’s stringent quality requirements [51]. The facility serves as a model for the adoption of European continuous manufacturing, with technology transfer programs supporting broader industry implementation.

Samsung BioLogics in South Korea has invested heavily in continuous manufacturing capabilities, establishing one of the world’s most extensive continuous bioprocessing facilities with capacity for multiple biosimilar products [53]. The facility demonstrates the economic viability of continuous manufacturing in emerging markets with significant cost advantages. The Asia–Pacific region exhibits the highest growth rate in the adoption of continuous manufacturing, driven by aggressive government support for biotechnology development and export competitiveness requirements [54].

## 4. Advanced Perfusion Cell Culture Technologies and Single-Use Systems

Perfusion cell culture represents the foundational technology enabling continuous upstream bioprocessing for recombinant protein production [7]. Unlike traditional fed-batch cell culture processes, which operate in discrete cycles with predetermined endpoint harvesting, perfusion processes maintain cells in a continuous, steady-state condition with ongoing nutrient supply and product removal [56].

### 4.1. Perfusion Process Fundamentals and Performance Characteristics

The perfusion process operates through a continuous exchange of cell culture medium, with fresh medium continuously fed into the bioreactor while spent medium and secreted products are continuously removed [57]. The critical enabling technology is the cell retention system, which maintains a viable cell population within the bioreactor while allowing for the continuous harvest of product-containing supernatant [58]. This approach enables cell densities exceeding 100 × 10^6^ cells/mL, compared to typical fed-batch densities of 10^6^–20 × 10^6^ cells/mL [59] (Table 7).

### 4.2. Cell Retention Technology Selection and Performance

The selection of appropriate cell retention technology represents a critical design decision that impacts overall perfusion system performance, scalability, and operational reliability [64]. Multiple cell retention approaches are available, each with distinct advantages and limitations that must be evaluated in the context of specific product and process requirements [65] (Table 8).

Tangential flow filtration systems utilize hollow fiber or flat sheet membrane configurations to achieve size-based separation between cells and product-containing medium [58]. TFF systems offer high retention efficiency and scalable operation but may introduce cell stress through recirculation pumping and are susceptible to membrane fouling, which can impact long-term operation [66]. Advanced TFF configurations, including alternating tangential flow systems, reduce cell stress through optimized flow patterns while maintaining high separation efficiency [67].

### 4.3. Single-Use Bioreactor Systems for Continuous Processing

Single-use bioreactor systems have emerged as critical enabling technologies for continuous bioprocessing, offering significant advantages in terms of flexibility, reduced contamination risk, and optimized capital costs [72]. The integration of single-use systems with perfusion technology creates powerful platforms for continuous manufacturing implementation (Table 9).

Single-use systems provide substantial economic benefits through the elimination of cleaning validation requirements, resulting in a 30–40% reduction in facility qualification costs by eliminating clean-in-place and steam-in-place requirements [77]. These systems enable 25–35% smaller facility requirements due to the elimination of cleaning utilities and storage, while providing rapid product changeover capabilities, enabling multi-product facilities [78]. The elimination of cross-contamination risks between products and batches represents a significant quality assurance advantage [79].

### 4.4. Integration with Downstream Processing

The successful implementation of perfusion cell culture requires careful integration with downstream processing operations to achieve end-to-end continuous manufacturing [80]. The continuous product stream from perfusion bioreactors must be compatible with downstream purification processes, requiring the coordination of flow rates, buffer compositions, and operational schedules between upstream and downstream operations [81]. Published case studies have demonstrated the successful integration of perfusion cell culture with continuous chromatography systems, achieving stable operation for 30+ days with consistent product quality and yield [62].

## 5. Continuous Chromatography Systems and Advanced Downstream Processing

**Figure 2:**
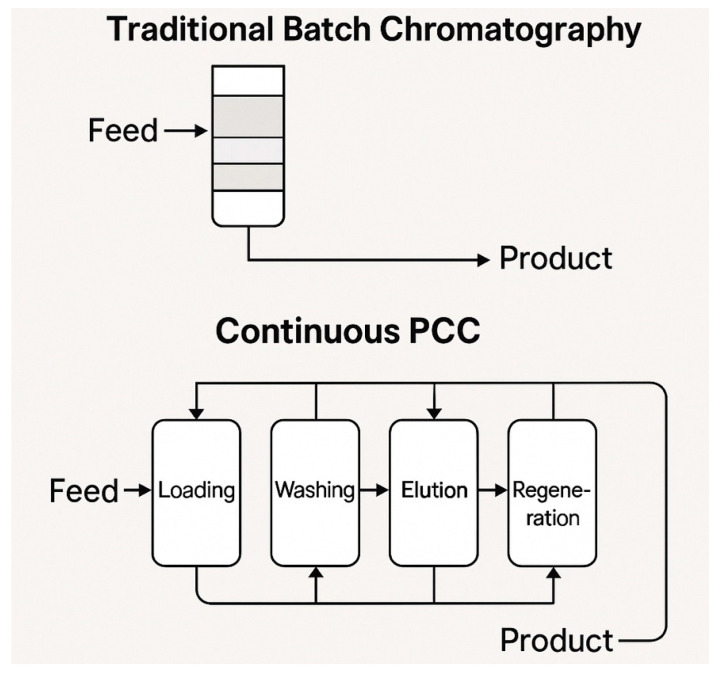
Process flow diagram comparing traditional batch chromatography with continuous PCC systems.

Continuous downstream processing represents the most technically challenging aspect of end-to-end continuous manufacturing for recombinant proteins [82]. Traditional downstream processing involves multiple discrete chromatography steps, each optimized independently and connected through intermediate storage and quality control testing [80] (Figure 2).

### 5.1. Periodic Counter-Current Chromatography Technology

Periodic counter-current chromatography has emerged as the leading technology for continuous protein capture chromatography, offering significant advantages over traditional single-column batch processes [83]. PCC systems utilize multiple chromatography columns operated in a synchronized manner, with columns cycling through loading, washing, elution, and regeneration phases while maintaining the continuous processing of the feed stream [84] (Table 10).

The operational principle of PCC involves strategically switching columns between different operational phases to maintain continuous loading capability while maximizing resin capacity utilization [89]. When the lead column approaches breakthrough, the feed stream is redirected to the next available column while the first column proceeds through the washing and elution phases [90]. This approach enables near-complete utilization of chromatography resin capacity compared to traditional batch processes that typically utilize only 60–80% of available capacity [85].

### 5.2. Multi-Column Continuous Chromatography Implementation

The implementation of multi-column continuous chromatography requires sophisticated process control and scheduling systems to coordinate column operations while maintaining consistent product quality [91]. The control system must manage valve switching sequences, flow rate coordination, buffer delivery timing, and quality monitoring across multiple columns operating in different phases simultaneously [92].

Published performance data demonstrate that PCC systems can achieve approximately 50% reduction in buffer consumption compared to traditional batch chromatography, corresponding to savings of 7400 L in a typical 20 kg monoclonal antibody clinical manufacturing campaign [87]. The buffer savings result from improved resin utilization and the elimination of the safety margins typically required in batch processes to prevent product breakthroughs [93].

### 5.3. Integrated Continuous Downstream Processing Platforms

Leading equipment manufacturers have developed integrated platforms that combine multiple unit operations in continuous mode, enabling comprehensive downstream processing solutions [94]. These platforms provide integrated hardware and software solutions for multi-column operation, including automated valve switching, real-time process monitoring, and product quality control capabilities [95] (Table 11).

## 6. Process Analytical Technology Implementation for Real-Time Quality Control

The successful implementation of continuous manufacturing for recombinant proteins requires sophisticated process analytical technology systems that provide real-time monitoring and control capabilities throughout the manufacturing process [99]. Unlike batch manufacturing, where quality control relies primarily on the offline testing of discrete samples, continuous manufacturing requires online and at-line analytical methods that provide immediate feedback for process control decisions without interrupting production flow [100] (Table 12).

### 6.1. Spectroscopic Methods for Real-Time Protein Monitoring

Near-infrared spectroscopy has emerged as a particularly valuable process analytical technology tool for continuous bioprocessing due to its ability to provide rapid, non-destructive analysis of multiple process parameters simultaneously [101]. NIR spectroscopy can monitor protein concentrations, cell density, metabolite concentrations, and product quality attributes in real time without requiring sample consumption or processing delays [102]. The implementation of NIR systems requires the development of robust chemometric models that correlate spectroscopic signals with relevant process parameters under varying operational conditions [111].

Raman spectroscopy offers complementary capabilities for real-time process monitoring, particularly for monitoring protein structural characteristics and aggregation states that may not be detectable through NIR spectroscopy [103]. Raman systems can be implemented with fiber-optic probes, enabling in situ monitoring within bioreactors and chromatography systems without the need for sample extraction or processing [104].

### 6.2. Online Quality Control Strategies and Digital Integration

The development of effective online quality control strategies for continuous manufacturing requires the integration of multiple analytical techniques to provide comprehensive process understanding and control capabilities [112]. Size exclusion chromatography systems can be implemented online to monitor protein aggregation and fragmentation in real time, providing critical quality information for process control decisions [107]. Mass spectrometry systems, while traditionally used for offline analysis, are increasingly being adapted for online implementation in continuous bioprocessing applications [108].

Modern PAT systems increasingly integrate with Industry 4.0 frameworks, enabling advanced data analytics, machine learning applications, and predictive process control [113]. The integration creates comprehensive digital ecosystems that enable real-time data analytics through advanced pattern recognition for the early detection of process deviations, multivariate statistical process control for monitoring complex parameters, and predictive modeling for proactive process adjustments [114,115,116].

### 6.3. Regulatory Considerations for PAT Implementation

Regulatory agencies have developed specific guidance for PAT implementation in continuous manufacturing, emphasizing the importance of method validation, calibration maintenance, and data integrity [117]. The FDA PAT Framework requires method validation protocols specific to continuous manufacturing applications, real-time release testing strategies with appropriate quality assurance, and data integrity requirements for electronic records and signatures [118,119,120]. The EMA Quality Guidelines address PAT method lifecycle management throughout commercial production, a risk-based approach to PAT implementation and validation, and harmonized inspection approaches for PAT-enabled continuous manufacturing [121,122,123].

## 7. Economic Analysis and Global Cost–Benefit Evaluation

The economic evaluation of continuous manufacturing implementation requires a comprehensive analysis of capital investment requirements, operational cost impacts, and revenue benefits across the entire product lifecycle [124]. Traditional financial analysis approaches may underestimate the full economic impact of continuous manufacturing due to the interconnected nature of benefits across multiple cost categories and the potential for operational improvements that may not be immediately apparent during initial implementation [125] (Table 13).

### 7.1. Capital Investment Analysis Across Global Regions

Regional variations in capital investment requirements reflect differences in labor costs, regulatory requirements, and the availability of infrastructure. The Asia–Pacific region demonstrates the highest cost reduction potential due to lower infrastructure costs and supportive government policies [128]. Capital investment requirements for continuous manufacturing implementation vary significantly depending on the specific technology choices, scale of implementation, and existing facility capabilities [130]. Greenfield continuous manufacturing facilities can potentially achieve a 30–50% reduction in capital costs compared to equivalent batch facilities due to fewer equipment requirements and reduced facility footprint [131] (Table 14).

### 7.2. Operational Cost Structure Analysis

The operational cost structure of continuous manufacturing differs substantially from traditional batch manufacturing across multiple categories [142]. Raw material consumption patterns change significantly, with continuous processes typically requiring higher media and buffer consumption rates offset by improved productivity and reduced waste generation [133]. A comprehensive lifecycle analysis is needed to accurately assess the net impact of these changes on overall manufacturing costs [143].

Labor cost impact varies depending on the degree of automation implemented in continuous manufacturing systems [144]. Highly automated continuous systems can achieve significant labor cost reductions by eliminating manual operations and reducing the need for supervision [135]. However, continuous systems require specialized technical expertise for maintenance and troubleshooting, which may require investment in employee training and development [145].

### 7.3. Return-on-Investment Analysis and Economic Modeling

Comprehensive economic modeling across multiple scenarios demonstrates favorable return-on-investment profiles for the implementation of continuous manufacturing [146]. The economic analysis includes payback periods of 3–5 years for greenfield implementations and 4–7 years for retrofits, net present value 15–25% higher than traditional batch investments over a 10-year horizon, internal rate of return of 18–28% depending on product portfolio and market conditions, and risk-adjusted returns showing continuous manufacturing demonstrates lower operational risk due to improved process control [147].

Equipment costs for continuous manufacturing systems are typically higher on a per-unit basis compared to traditional batch equipment due to the increased complexity and specialized nature of continuous processing technologies [148]. Perfusion bioreactor systems may cost 50–100% more than equivalent fed-batch systems due to the additional complexity of cell retention systems and perfusion control equipment [149]. Similarly, multi-column chromatography systems require substantially higher initial investment compared to single-column batch systems [150].

## 8. Global Regulatory Strategy Development and Implementation Approaches

### 8.1. Regulatory Pathway Analysis and Submission Requirements

The regulatory pathway for the continuous manufacturing of recombinant drugs necessitates the development of a comprehensive strategy that addresses the unique challenges associated with demonstrating equivalence to existing batch processes, while leveraging the enhanced process understanding and control capabilities that continuous manufacturing enables [151]. Regulatory agencies worldwide have invested substantial effort in developing guidance and expertise to support the implementation of continuous manufacturing. Yet, manufacturers must still navigate complex submission requirements and demonstrate robust process control capabilities [152] (Table 15).

### 8.2. Regional Regulatory Harmonization Initiatives

The Asia-Pacific Economic Cooperation has established working groups for continuous manufacturing regulatory harmonization, focusing on mutual recognition agreements and shared inspection protocols [160]. Japan’s PMDA leads regional coordination efforts, with Singapore’s HSA and Australia’s TGA participating in pilot programs for harmonized continuous manufacturing evaluation [161]. The EMA has established a specialized Continuous Manufacturing Assessment Team comprising experts from member state regulatory agencies [155]. The team has developed standardized assessment procedures and training modules for continuous manufacturing evaluation across all EU member states [162].

The Pan American Network for Drug Regulatory Harmonization has initiated collaborative programs for the evaluation of continuous manufacturing, with a particular focus on biosimilar applications to improve healthcare access in Latin American markets [163]. These regional harmonization efforts facilitate technology transfer and implementation across diverse regulatory environments while maintaining appropriate quality standards [164].

### 8.3. Pre-Submission Engagement and Strategic Approaches

Regulatory agencies worldwide have established formal pre-submission pathways for continuous manufacturing applications, offering manufacturers opportunities for early feedback and risk mitigation [165]. The FDA Emerging Technology Program has conducted over 27 meetings with companies regarding continuous manufacturing, utilizing specialized review teams with enhanced expertise in continuous manufacturing and offering fast-track designation opportunities for breakthrough manufacturing technologies [166,167,168].

The EMA Scientific Advice program offers dedicated consultation pathways for continuous manufacturing, multi-stakeholder meetings that include academic and industry experts, and harmonized advice across EU member states [169,170,171]. These pre-submission engagement opportunities enable manufacturers to address potential regulatory concerns early in the development process and align their implementation strategies with regulatory expectations [172].

## 9. Implementation Challenges and Advanced Risk Mitigation Strategies

### 9.1. Technology Integration Complexity and System Design

The integration of multiple continuous unit operations into cohesive manufacturing systems presents significant technical challenges that require sophisticated engineering approaches and comprehensive system design methodologies [173]. Unlike batch processes, where individual unit operations can be optimized independently, continuous manufacturing requires coordinated optimization across all integrated processes to achieve stable system operation [174] (Table 16).

Flow rate balancing between upstream and downstream operations represents a fundamental challenge in implementing continuous manufacturing, particularly given the differing operational characteristics and capacity constraints of various unit operations [182]. Perfusion bioreactors can operate at steady flow rates for extended periods, whereas continuous chromatography systems often require variable flow rates during different operational phases [183]. Advanced process control systems must coordinate these varying requirements while maintaining overall system stability [184].

### 9.2. Process Development and Scale-Up Considerations

The process development paradigm for continuous manufacturing differs fundamentally from traditional batch development approaches, requiring new methodologies and experimental strategies to characterize process behavior and optimize performance [177]. Traditional scale-up approaches based on geometric similarity and dimensionless number scaling may not be directly applicable to continuous processes, where residence time distributions and mixing characteristics significantly impact performance [178].

Process validation for continuous manufacturing presents unique challenges compared to traditional batch validation approaches, requiring new methodologies and regulatory acceptance criteria [185]. Process Performance Qualification requires extended campaigns demonstrating consistent operation, real-time release testing validation of PAT methods for quality assessment, material diversion systems validation of out-of-specification material handling, and lifecycle management ongoing validation throughout commercial production [186,187,188,189].

### 9.3. Organizational Change Management and Workforce Development

The implementation of continuous manufacturing requires significant organizational change management efforts to address cultural adaptation, training requirements, and capability development [179]. Organizations must invest in training and capability development across multiple disciplines, including process engineering, advanced analytics, automation systems, and regulatory sciences [180]. The interdisciplinary nature of continuous manufacturing necessitates collaboration across traditional organizational boundaries and may require modifications to the organizational structure to support integrated process management [190].

Successful implementation requires comprehensive change management strategies that address leadership commitment and strategic alignment, cultural transformation, and the adoption of a continuous improvement mindset. Additionally, it entails capability development, investment in training and expertise building, and technology partnerships and collaboration with equipment vendors and technology providers [191,192,193,194].

## 10. Future Technology Evolution and Industry 4.0 Integration

### 10.1. Emerging Technologies for Continuous Manufacturing Enhancement

The continuous manufacturing landscape for recombinant drugs is rapidly evolving, with emerging technologies promising to enhance the advantages of continuous processing further while addressing current implementation challenges [195]. These developments span multiple areas, including advanced automation technologies, artificial intelligence applications, novel process intensification approaches, and integrated digital manufacturing platforms [196] (Table 17).

The integration of artificial intelligence and machine learning technologies represents a transformative opportunity for continuous manufacturing optimization that extends far beyond traditional process control approaches [197]. Current applications of AI/ML in continuous manufacturing primarily focus on process monitoring and fault detection. Still, emerging applications include predictive process optimization, automated process development, and autonomous operation of manufacturing systems [198].

### 10.2. Industry 4.0 Implementation Roadmap and Digital Transformation

The integration of continuous manufacturing with Industry 4.0 technologies creates opportunities for unprecedented levels of automation, optimization, and quality assurance [207]. The digital transformation strategy encompasses four implementation phases: basic digital integration with existing PAT systems in years 1–2, advanced analytics and machine learning implementation in years 2–4, autonomous operation and predictive optimization in years 4–6, and whole Industry 4.0 integration with supply chain networks in years 6–8 [208] (Table 18).

Machine learning algorithms trained on comprehensive process datasets can identify complex relationships between process parameters and product quality that may not be apparent through traditional process understanding approaches [214]. These algorithms can continuously learn from process operation data, identifying optimization opportunities and predicting process performance under varying operational conditions [215].

### 10.3. Cybersecurity and Data Integrity Frameworks

The increasing digitalization of continuous manufacturing systems necessitates robust cybersecurity frameworks to safeguard critical manufacturing infrastructure and maintain data integrity [216]. The cybersecurity implementation requirements include network segmentation for the essential isolation of manufacturing systems, access control through multi-factor authentication and role-based permissions, data encryption for the protection of intellectual property and process data, and incident response with rapid response protocols for cybersecurity threats [217,218,219,220].

Advanced cybersecurity frameworks must address the unique vulnerabilities introduced by continuous manufacturing systems, including the integration of operational technology with information technology networks, the real-time data communication requirements that may compromise traditional security measures, and the potential for cyberattacks to impact product quality and patient safety [221]. Regulatory agencies are developing specific guidance for cybersecurity requirements in continuous manufacturing environments, emphasizing the importance of risk-based approaches to cybersecurity implementation [222].

## 11. Supply Chain Integration and Logistics Optimization

### 11.1. Continuous Manufacturing Supply Chain Transformation

The implementation of continuous manufacturing fundamentally changes supply chain requirements, demanding new approaches to raw material management, finished product distribution, and logistics coordination [223]. Traditional batch manufacturing supply chains are designed around discrete production campaigns with large inventory buffers, while continuous manufacturing requires just-in-time coordination and minimal inventory holdings [224] (Table 19).

Raw material management in continuous manufacturing requires sophisticated coordination between suppliers and manufacturers to ensure consistent quality and timely delivery [225]. The reduction in inventory levels from 60 to 80% compared to batch manufacturing creates significant working capital improvements but requires enhanced supplier reliability and quality assurance programs [230]. Quality control transformation from batch release testing to real-time quality monitoring eliminates hold times and accelerates product release but requires extensive method validation and regulatory acceptance [226].

### 11.2. Digital Supply Chain Integration and Advanced Technologies

Advanced supply chain technologies enable the coordination and optimization necessary for the successful implementation of continuous manufacturing [231]. Predictive analytics provide demand forecasting and capacity planning optimization, allowing manufacturers to align production rates with market demand while minimizing inventory holdings [232]. Blockchain integration ensures end-to-end traceability and supply chain transparency, addressing regulatory requirements for material genealogy in continuous manufacturing [233].

IoT-enabled logistics provide real-time tracking and environmental monitoring throughout the supply chain, ensuring the maintenance of product quality during transportation and distribution [234]. Automated inventory management through AI-driven inventory optimization reduces manual oversight requirements while maintaining appropriate stock levels for continuous operation [235] (Table 20).

### 11.3. Cost Analysis and Economic Impact of Integrated Supply Chains

The economic impact of supply chain integration extends beyond direct cost savings to include improved cash flow through reduced working capital requirements and enhanced customer service through shorter lead times and improved product availability [241]. The transformation requires significant investment in digital infrastructure and the development of supplier capabilities but provides substantial long-term competitive advantages [242].

## 12. Global Healthcare Access and Societal Impact

### 12.1. Healthcare Access Enhancement Through Manufacturing Cost Reduction

The widespread adoption of continuous manufacturing for recombinant drugs has the potential to fundamentally transform global healthcare access through dramatic reductions in manufacturing costs and corresponding improvements in drug affordability [243]. The impact extends beyond simple cost reduction to encompass increased manufacturing capacity, improved supply chain resilience, and enhanced ability to respond to emerging healthcare needs [244] (Table 21).

The cost reduction potential of continuous manufacturing implementation creates opportunities for significant healthcare cost savings across multiple categories of biological therapeutics [243]. Current projections suggest that biosimilars enabled by continuous manufacturing technologies could generate savings of USD 125–237 billion between 2023 and 2027 in the United States alone, with individual patients potentially saving USD 1800–5500 annually through increased access to cost-effective biological therapeutics [43,44].

### 12.2. Technology Transfer and Economic Development Opportunities

Continuous manufacturing enables cost-effective production at smaller scales, making biological therapeutics economically viable for smaller patient populations and emerging markets where traditional large-scale batch manufacturing may not be economically justified [250]. Technology transfer programs facilitate the implementation of continuous manufacturing in developing markets through WHO Prequalification with expedited pathways for continuous manufacturing facilities in developing countries, academic partnerships for university–industry collaboration for technology transfer, government support through public–private partnerships for healthcare access improvement, and philanthropic initiatives with foundation-supported implementation programs [251,252,253,254].

The economic development impact extends beyond healthcare to include job creation in high-technology manufacturing, the development of technical expertise and capabilities, the attraction of foreign investment in biotechnology sectors, and the establishment of regional manufacturing hubs for biological therapeutics [255]. These broader economic benefits justify government support for the implementation of continuous manufacturing and technology transfer programs [256].

### 12.3. Environmental Sustainability and Impact Assessment

Continuous manufacturing offers significant environmental advantages, including reduced resource consumption, waste generation, and energy utilization, compared to traditional batch manufacturing [257]. The environmental benefits include substantial reductions in water consumption, energy consumption, waste generation, chemical consumption, and carbon footprint across the manufacturing lifecycle [258] (Table 22).

The environmental benefits of continuous manufacturing align with increasing regulatory and societal pressure for sustainable manufacturing practices [264]. Many regulatory agencies now offer incentives for environmentally sustainable manufacturing approaches, including expedited review pathways and reduced fees for manufacturers that demonstrate significant environmental improvements [265]. The alignment of economic and ecological benefits creates compelling business cases for the adoption of continuous manufacturing [266].

## 13. Strategic Implementation Recommendations and Future Outlook

### 13.1. Comprehensive Implementation Roadmap and Strategic Planning

Organizations considering the implementation of continuous manufacturing should adopt phased implementation strategies that build capabilities progressively while managing implementation risks [20]. The strategic approach must balance technological complexity, regulatory requirements, economic considerations, and organizational readiness to ensure successful adoption [267] (Table 23).

Initial deployments should focus on well-characterized products with established market demand and robust technical understanding to minimize technical and commercial risks during the learning curve period [268]. The development of internal technical capabilities is a critical success factor for implementing continuous manufacturing, requiring organizations to invest in training and capability development across multiple disciplines [269].

### 13.2. Critical Success Factors and Implementation Best Practices

Successful continuous manufacturing implementation requires alignment across multiple organizational dimensions and sustained commitment to transformation initiatives [276]. Organizational readiness factors include leadership commitment and strategic alignment, cultural transformation and adoption of a continuous improvement mindset, capability development and investment in training and expertise building, and technology partnerships and collaboration with equipment vendors and technology providers [277,278,279,280].

Technical excellence requirements encompass process understanding and a deep knowledge of product and process requirements, quality systems, and robust quality management and control strategies, as well as regulatory strategies and proactive engagement with regulatory agencies. Additionally, risk management is ensured through comprehensive risk assessment and mitigation strategies [281,282,283,284]. The interdisciplinary nature of continuous manufacturing necessitates collaboration across traditional organizational boundaries and may require modifications to organizational structure to support integrated process management [285].

### 13.3. Future Market Evolution and Growth Opportunities

The continuous manufacturing market for biopharmaceuticals is projected to grow at a compound annual growth rate of 15.9% through 2030, driven by cost reduction pressures, regulatory support, and technological maturation [286]. Market growth is expected to be particularly strong in emerging markets, where improvements in healthcare access create substantial demand for cost-effective biological therapeutics [287] (Table 24).

The convergence of continuous manufacturing with other emerging technologies creates additional opportunities for innovation and competitive advantage [293]. Technology convergence areas include AI-enabled bioprocessing for machine learning optimization of biological systems, personalized medicine manufacturing for small-scale, patient-specific production, distributed manufacturing networks for regional production capabilities, and sustainable bioprocessing for minimizing environmental impact [294,295,296,297].

### 13.4. Long-Term Vision and Industry Transformation

The trajectory of continuous manufacturing development indicates continued technology advancement and expanding industry adoption over the next decade [298]. The convergence of continuous manufacturing with artificial intelligence, advanced automation, digital manufacturing technologies, and sustainability initiatives promises to enhance the advantages of continuous processing further while addressing current implementation challenges and creating new opportunities for pharmaceutical innovation and global healthcare improvement [275].

The long-term vision for continuous manufacturing encompasses the establishment of distributed manufacturing networks optimized for regional markets, the development of autonomous manufacturing systems requiring minimal human intervention, the integration of sustainability principles throughout the manufacturing lifecycle, and the democratization of biological therapeutic manufacturing to improve global healthcare access [299,300,301,302]. This transformation will require sustained investment in technology development, regulatory harmonization, and capability building across the global pharmaceutical industry [303].

## 14. Conclusions

The comprehensive analysis presented in this review demonstrates that the continuous manufacturing of recombinant drugs represents a fundamental paradigm shift in biopharmaceutical manufacturing, offering compelling technical, economic, and strategic advantages compared to traditional batch manufacturing approaches [304]. The evidence from validated case studies, economic analyses, regulatory developments, and global implementation experiences indicates that continuous manufacturing has evolved from an experimental concept to a commercially viable manufacturing strategy with growing industry adoption and regulatory support worldwide [305].

The technical foundations for the continuous manufacturing of recombinant drugs have reached sufficient maturity to support commercial implementation across a wide range of product types, manufacturing scales, and geographic regions [298]. Perfusion cell culture technologies have demonstrated consistent operation for extended periods, exceeding 60 days, with stable productivity and product quality. In contrast, multi-column continuous chromatography systems have achieved commercial-scale implementation, demonstrating buffer savings of 50% and improved resin utilization [62,87]. Process analytical technology systems have evolved to provide comprehensive real-time monitoring and control capabilities, enabling robust process control throughout extended continuous operation periods [306].

The economic analysis demonstrates that continuous manufacturing provides significant value creation opportunities across multiple dimensions, including capital investment optimization, operational cost reduction, and enhanced asset utilization [307]. The potential for a 30–50% reduction in facility capital requirements through process intensification and integration represents substantial value creation opportunities for both new facility construction and existing facility optimization, with regional variations reflecting local economic conditions and regulatory requirements [131]. The global healthcare access implications of widespread continuous manufacturing adoption extend far beyond simple cost reduction to encompass improved availability of biological therapeutics in underserved markets, enhanced supply chain resilience, and accelerated response capabilities for emerging healthcare needs [243,244].

The establishment of the ICH Q13 framework represents a watershed moment in pharmaceutical regulatory harmonization, providing the foundation for consistent global implementation of continuous manufacturing technologies [13]. The coordinated adoption across major regulatory jurisdictions, including specialized implementation programs and enhanced reviewer training, demonstrates unprecedented international collaboration in support of manufacturing innovation [166]. Regional adaptation of ICH Q13 guidance has proceeded smoothly, with specialized programs in the Asia–Pacific, Latin America, and other emerging markets creating pathways for technology transfer and local implementation [160,163].

Organizations considering the implementation of continuous manufacturing should adopt phased implementation strategies that build capabilities progressively while managing implementation risks [20]. The strategic approach must strike a balance between technological complexity, regulatory requirements, economic considerations, and organizational readiness to ensure successful adoption [267]. Successful implementation requires alignment across multiple organizational dimensions and sustained commitment to transformation initiatives, including leadership commitment, technical excellence, and strategic partnerships [276].

The trajectory of continuous manufacturing development indicates continued technology advancement and expanding industry adoption over the next decade [298]. The convergence of continuous manufacturing with artificial intelligence, advanced automation, digital manufacturing technologies, and sustainability initiatives promises to enhance the advantages of continuous processing further while addressing current implementation challenges and creating new opportunities for pharmaceutical innovation and global healthcare improvement [275]. This transformation represents not only technological evolution but also a fundamental shift toward more sustainable, accessible, and cost-effective biopharmaceutical manufacturing, which will benefit patients worldwide [308].

## Figures and Tables

**Table 1:** Historical development of manufacturing approaches in related industries.

Industry	First Implementation	Key Technology	References
Chemical Industry	Early 19th century	Sulfuric acid continuous production	[4]
Petrochemical	1920	Continuous ethane to ethylene conversion	[5]
Pharmaceutical	2010s	Continuous tablet manufacturing	[6]
Biopharmaceutical	2015–present	Perfusion-based continuous processing	[7]

**Table 2:** ICH Q13 guidance structure and content overview.

Component	Pages	Content Focus	Key Requirements	References
Main Guidance	15	Fundamental principles, development approaches	Enhanced process understanding	[13]
Annex I	4	Small molecule continuous manufacturing	Process control strategies	[13]
Annex II	6	Drug product continuous manufacturing	Material diversion systems	[13]
Annex III	8	Therapeutic protein drug substances	Biological system considerations	[16]
Annex IV	3	Quality considerations	Real-time monitoring	[13]
Annex V	3	Regulatory submission guidance	Documentation requirements	[13]

**Table 3:** International regulatory implementation timeline and regional adaptations.

Region/Agency	Implementation Date	Special Initiatives	Regional Adaptations	References
FDA (United States)	March 2023	Emerging Technology Program	Fast-track pathways for continuous manufacturing	[26]
EMA (Europe)	July 2023	Implementation Working Group	Centralized review procedures	[26]
Health Canada (Canada)	September 2023	Parallel review processes	Mutual recognition with the FDA	[27]
PMDA (Japan)	October 2023	Technical guidance adaptation	Asia–Pacific harmonization	[28]
NMPA (China)	January 2024	Pilot program initiative	Emerging technology pathways	[29]
ANVISA (Brazil)	April 2024	Biosimilar focus program	PANDRH collaboration	[30]

**Table 4:** Economic pressures are driving the adoption of continuous manufacturing.

Factor	Impact	Cost Range	References
Average biotechnology drug development cost	USD 1.9 billion (2012)	Higher estimates for recent years	[10]
Market value of US biologics (2024)	USD 487 billion	Annual expenditure	[11]
Traditional facility capital investment	USD 500 million–2 billion	Depending on capacity/complexity	[36]
Continuous manufacturing facility investment	USD 100 million–300 million	Reduced capital requirements	[37]

**Table 5:** Global biosimilar market impact and regional penetration.

Metric	Value	Period	Regional Distribution	References
Global biosimilar contract manufacturing market (2023)	USD 8.59 billion	2023 baseline	45% Europe, 30% North America, 25% Asia–Pacific	[42]
Projected CAGR	15.9%	2024–2030	Asia–Pacific: 18.2%; Europe: 14.8%; North America: 13.5%	[42]
US biosimilar savings projection	USD 125–237 billion	2023–2027	Federal programs: 40%; private payers: 60%	[43,44]
EU biosimilar market penetration	35% average	2024	Range: 15% (France) to 80% (Denmark)	[45]

**Table 6:** Regional continuous manufacturing adoption patterns and leading implementations.

Region	Implementation Status	Key Drivers	Market Penetration	Leading Companies	Notable Facilities	References
North America	Commercial scale	Cost reduction, FDA support	25% of new facilities	Genentech, Amgen, Pfizer	Genentech South San Francisco	[49,50]
Europe	Rapid adoption	EMA harmonization, cost pressures	30% of new facilities	Novartis, Roche, Biogen	Biogen Denmark facility	[51,52]
Asia–Pacific	Aggressive growth	Export competitiveness	35% of new facilities	Samsung, WuXi, Celltrion	Samsung BioLogics Korea	[53,54]
Latin America	Early stage	Healthcare access, cost reduction	10% of new facilities	Biosidus, Probiomed	Regional pilot programs	[55]

**Table 7:** Performance comparison between fed-batch and perfusion cell culture systems.

Parameter	Fed-Batch	Perfusion	Improvement Factor	References
Cell density (cells/mL)	10^6^–20 × 10^6^	>100 × 10^6^	5–10×	[59]
Volumetric productivity	Baseline	3–5× higher	3–5×	[60]
Product residence time	10–14 days	1–3 days	3–5× reduction	[61]
Continuous operation period	N/A	>60 days	Sustained	[62]
Bioreactor volume requirement	15,000–25,000 L	1000–2000 L	70% reduction	[63]

N/A: Not Applicable.

**Table 8:** Cell retention technologies for perfusion systems with commercial availability.

Technology	Separation Principle	Advantages	Limitations	Commercial Vendors	References
Tangential Flow Filtration (TFF)	Size-based membrane separation	High retention efficiency, scalable	Membrane fouling, cell stress	Cytiva, Merck KGaA	[66]
Alternating Tangential Flow (ATF)	Optimized TFF with reduced stress	Reduced cell stress, high efficiency	Complex operation	Repligen Corporation	[67]
Acoustic Wave Separation (AWS)	Ultrasonic cell aggregation	Gentle handling, no fouling	Limited commercial scale	FloDesign Sonics	[68,69]
Centrifugal Separation	Gravitational separation	High capacity, robust	Cell stress from forces	Pneumatically Integrated	[70,71]

**Table 9:** Single-use perfusion bioreactor systems with specifications and features.

Vendor	System	Scale Range	Key Features	Perfusion Integration	Economic Benefits	References
Cytiva (Marlborough, MA, USA)	Xcellerex XDR	50–2000 L	Integrated control, disposable sensors	Native ATF integration	30% validation cost reduction	[73]
Thermo Fisher (Waltham, MA, USA)	HyPerforma DynaDrive	50–1000 L	Dynamic impeller, advanced mixing	TFF-ready design	25% facility footprint reduction	[74]
Sartorius (Göttingen, Germany)	BIOSTAT STR	50–2000 L	Stirred tank, flexible configuration	Modular perfusion options	Rapid product changeover	[75]
Eppendorf (Hamburg, Germany)	BioFlo 720	1–50 L	Compact design, parallel processing	Research-scale perfusion	Contamination risk elimination	[76]

**Table 10:** PCC performance benefits and commercial system specifications.

Parameter	Batch Process	PCC Process	Improvement	Commercial System	Vendor	References
Resin capacity utilization	60–80%	90–95%	15–35% increase	ÄKTA pcc 75	Cytiva	[85,86]
Buffer consumption	Baseline	50% reduction	50% savings	Contichrom CUBE	ChromaCon	[87,88]
Processing efficiency	Single column	Multi-column continuous	Continuous flow	CaptureSMB	GE Healthcare	[84]
Buffer savings (20 kg mAb campaign)	Baseline	7400 L saved	Significant reduction	BioSMB Platform	Multiple vendors	[87]

**Table 11:** Integrated continuous downstream processing platforms and capabilities.

Platform	Vendor	Unit Operations	Capacity Range	Integration Level	Key Features	References
ÄKTA process	Cytiva	Capture, polishing, UF/DF	1–100 kg/batch	Fully integrated	Automated control, PAT integration	[96]
ChromaCon CUBE	ChromaCon	Multi-column chromatography	Pilot to commercial	Modular integration	Real-time monitoring, flexible configuration	[97]
OPUS platform	Merck KGaA	Continuous processing suite	Research to production	Platform approach	Scalable design, digital integration	[98]

**Table 12:** Comprehensive PAT technologies for continuous bioprocessing applications.

Technology	Application	Parameters Monitored	Advantages	Implementation Complexity	Cost Range	References
Near-Infrared (NIR)	Real-time protein monitoring	Concentration, cell density, metabolites	Non-destructive, rapid analysis	Moderate	USD 50 kilo–200 kilo	[101,102]
Raman Spectroscopy	Structural analysis	Protein structure, aggregation	In situ probes available	Moderate	USD 75 kilo–300 kilo	[103,104]
Mid-Infrared (MIR)	Detailed protein analysis	Structure, modifications	High specificity	High	USD 100 kilo–400 kilo	[105,106]
Online SEC	Quality monitoring	Aggregation, fragmentation	Real-time quality data	High	USD 150 kilo–500 kilo	[107]
Mass Spectrometry	Comprehensive analysis	Modifications, impurities	Detailed characterization	Very High	USD 300 kilo–1 million	[108,109]
Fluorescence	Cell viability monitoring	Viable cell density, metabolism	Rapid response	Low	USD 25 kilo–100 kilo	[110]

**Table 13:** Regional capital investment comparison for continuous manufacturing facilities.

**Region**	**Traditional Billionatch Facility**	**Continuous Facility**	**Cost Reduction**	**Productivity Gain**	**Payback Period**	**Reference** **s**
North America	USD 800 million–1.5 billion	USD 400 million–900 million	40–50%	3–4×	3–5 years	[126]
Europe	USD 700 million–1.2 billion	USD 350 million–750 million	35–45%	2.5–3.5×	4–6 years	[127]
Asia–Pacific	USD 500 million–900 million	USD 250 million–500 million	45–55%	3–5×	3–4 years	[128]
Latin America	USD 300 million–600 million	USD 150 million–350 million	40–50%	2–4×	4–7 years	[129]

**Table 14:** Comprehensive operational cost analysis by category and region.

Cost Category	Traditional Batch	Continuous Manufacturing	Cost Impact	Regional Variation	References
Raw materials (% of total)	15–25%	12–20%	15–25% reduction	Asia: higher savings	[132,133]
Labor costs (% of total)	20–30%	12–20%	25–40% reduction	Europe: moderate savings	[134,135]
Quality control	High offline testing	Reduced with PAT	30–50% reduction	Global consistent	[136,137]
Energy consumption	Baseline	Integrated efficiency	15–25% reduction	Variable by region	[138,139]
Facility utilization	60–70%	85–95%	20–35% improvement	Consistent globally	[140]
Maintenance costs	Scheduled downtime	Predictive maintenance	20–30% reduction	Technology dependent	[141]

**Table 15:** Global regulatory pathway comparison and implementation requirements.

Region	Primary Guidance	Submission Timeline	Special Requirements	Review Duration	Success Rate	References
United States (FDA)	ICH Q13 + FDA Guidance	Standard BLA/NDA pathway	Emerging Technology Program	10–12 months	85%	[153,154]
Europe (EMA)	ICH Q13 + EMA Guidelines	Centralized procedure	Scientific advice meetings	12–15 months	80%	[155]
Japan (PMDA)	ICH Q13 + J-GMP adaptation	Standard pathway	Prior consultation	12–14 months	78%	[156]
China (NMPA)	ICH Q13 + local requirements	Priority review pathway	Technical review meetings	8–12 months	70%	[157]
Canada (Health Canada)	ICH Q13 + Canadian guidance	Parallel FDA review	Mutual recognition protocols	10–13 months	82%	[158]
Brazil (ANVISA)	ICH Q13 + local adaptation	Accelerated pathway	Biosimilar focus program	12–18 months	65%	[159]

**Table 16:** Implementation challenges and comprehensive mitigation strategies.

Challenge Category	Specific Issues	Risk Level	Mitigation Strategies	Success Factors	Implementation Timeline	References
Technology Integration	Flow rate balancing, system coordination	High	Advanced process control, phased implementation	Cross-functional teams	12–18 months	[173,174]
Material Tracking	Continuous flow traceability	Medium	Residence time modeling, statistical tracking	Digital integration	6–12 months	[175,176]
Process Development	Scale-up methodology differences	Medium	Model-based approaches, extended characterization	Regulatory alignment	18–24 months	[177,178]
Organizational Change	Training, cultural adaptation	High	Change management, skills development	Leadership commitment	24–36 months	[179,180]
Regulatory Compliance	Validation complexity	Medium	Early agency engagement, robust documentation	Proactive strategy	12–24 months	[151,152]
Supply Chain Integration	Just-in-time coordination	Medium	Digital supply networks, predictive analytics	Supplier partnerships	12–18 months	[181]

**Table 17:** Emerging technologies and implementation timelines for continuous manufacturing.

Technology Area	Current Applications	Future Potential	Implementation Timeline	Investment Level	Expected ROI	References
Artificial Intelligence/ML	Process monitoring, fault detection	Autonomous operation, predictive optimization	2–5 years	High	25–40%	[197,198]
Advanced Robotics	Automated sampling, maintenance	Fully autonomous manufacturing	3–7 years	Very High	30–50%	[199,200]
Process Intensification	Microfluidics, novel bioreactors	Dramatically reduced footprints	5–10 years	Medium	20–35%	[201,202]
Modular Systems	Plug-and-play components	Rapid product changeover	2–5 years	Medium	15–30%	[203,204]
Digital Twins	Process simulation	Predictive optimization	1–3 years	Medium	20–35%	[205]
Blockchain	Supply chain tracking	End-to-end traceability	3–5 years	Low	10–20%	[206]

**Table 18:** Industry 4.0 technologies and continuous manufacturing applications.

Technology	Application	Benefits	Implementation Complexity	ROI Timeline	Current Adoption	References
IoT Sensors	Real-time monitoring	Comprehensive data collection	Low	1–2 years	60% industry adoption	[209]
Edge Computing	Local data processing	Reduced latency, improved control	Medium	2–3 years	35% industry adoption	[210]
Cloud Analytics	Big data analysis	Predictive insights	Medium	2–4 years	45% industry adoption	[211]
Digital Twins	Process simulation	Optimization, risk reduction	High	3–5 years	20% industry adoption	[212]
AI/ML Platforms	Autonomous control	Self-optimizing processes	Very High	4–7 years	15% industry adoption	[213]

**Table 19:** Supply chain transformation requirements for continuous manufacturing implementation.

Supply Chain Element	Traditional Batch	Continuous Manufacturing	Key Changes	Implementation Challenges	Cost Impact	References
Raw Material Management	Bulk delivery, large inventory	Just-in-time delivery, small inventory	60–80% inventory reduction	Supply reliability, quality assurance	30–50% cost reduction	[225]
Quality Control	Batch release testing	Real-time quality monitoring	Elimination of hold times	Method validation, regulatory acceptance	40–60% cost reduction	[226]
Finished Product	Large batch releases	Continuous product flow	Improved cash flow	Distribution network redesign	20–35% improvement	[227]
Cold Chain Management	Batch-based logistics	Continuous flow requirements	Temperature consistency	Infrastructure investment	Variable impact	[228]
Supply Network Design	Hub-and-spoke model	Distributed manufacturing	Regional production capabilities	Technology transfer complexity	25–45% cost reduction	[229]

**Table 20:** Supply chain cost comparison between batch and continuous manufacturing.

Cost Category	Batch Manufacturing	Continuous Manufacturing	Cost Impact	Regional Variation	Implementation Timeline	References
Inventory carrying costs	8–12% of product value	2–4% of product value	60–75% reduction	Consistent globally	6–12 months	[236]
Transportation costs	High, batch-based	Optimized, continuous flow	20–35% reduction	Higher in remote regions	12–18 months	[237]
Warehouse requirements	Large, batch storage	Minimal, flow-through	70–85% reduction	Variable by infrastructure	18–24 months	[238]
Quality control costs	High, batch testing	Reduced, real-time monitoring	40–60% reduction	Technology dependent	12–24 months	[239]
Working capital	High inventory investment	Low inventory investment	50–70% improvement	Cash flow benefits	6–18 months	[240]

**Table 21:** Healthcare access impact projections by global region.

Region	Current Access Level	Projected Improvement	Cost Reduction Target	Patient Impact	Implementation Timeline	References
North America	85% coverage	5–10% improvement	30–40% cost reduction	2 M additional patients	5–7 years	[245]
Europe	90% coverage	3–7% improvement	25–35% cost reduction	1.5 M additional patients	4–6 years	[246]
Asia–Pacific	60% coverage	15–25% improvement	40–55% cost reduction	50 M additional patients	7–10 years	[247]
Latin America	40% coverage	20–35% improvement	45–60% cost reduction	25 M additional patients	8–12 years	[248]
Africa	25% coverage	30–50% improvement	50–70% cost reduction	100 M additional patients	10–15 years	[249]

**Table 22:** Environmental impact comparison between manufacturing approaches.

Environmental Factor	Batch Manufacturing	Continuous Manufacturing	Improvement	Global Impact	Regulatory Recognition	References
Water consumption	100,000–500,000 L/kg	30,000–150,000 L/kg	60–70% reduction	Water conservation	EPA/EMA sustainability guidelines	[259]
Energy consumption	Baseline	15–25% reduction	Energy efficiency	Carbon footprint reduction	Green manufacturing incentives	[260]
Waste generation	High solvent usage	Reduced through integration	40–60% reduction	Waste minimization	Waste reduction regulations	[261]
Chemical consumption	Large buffer volumes	Optimized usage	30–50% reduction	Environmental protection	Chemical safety guidelines	[262]
Carbon footprint	High energy intensity	Optimized processes	20–35% reduction	Climate change mitigation	Carbon tax advantages	[263]

**Table 23:** Strategic implementation phases and comprehensive success factors.

Implementation Phase	Duration	Key Activities	Success Metrics	Investment Level	Risk Level	References
Phase 1: Assessment	6–12 months	Technology evaluation, capability assessment	Internal expertise development	Low (USD 1 million–5 million)	Low	[268,269]
Phase 2: Pilot Implementation	12–18 months	Small-scale demonstration, proof of concept	Technical feasibility demonstration	Medium (USD 5 million–25 million)	Medium	[270,271]
Phase 3: Scale-up	18–36 months	Commercial implementation, process optimization	Regulatory approval, commercial production	High (USD 25 million–100 million)	High	[272,273]
Phase 4: Expansion	3–5 years	Multi-product implementation, global rollout	Market penetration, competitive advantage	Very High (USD 100 million+)	Medium	[274,275]

**Table 24:** Future market opportunities and growth projections by therapeutic segment.

Market Segment	Current Size (2024)	Projected 2030 Size	CAGR	Key Growth Drivers	Continuous Manufacturing Impact	References
Monoclonal Antibodies	USD 185 billion	USD 425 billion	12.8%	Biosimilar competition, cost pressures	High cost reduction potential	[288]
Recombinant Proteins	USD 85 billion	USD 180 billion	11.2%	Emerging markets, accessibility	Manufacturing scalability	[289]
Gene Therapies	USD 15 billion	USD 65 billion	23.5%	Technology advancement, regulatory support	Production cost reduction	[290]
Cell Therapies	USD 8 billion	USD 45 billion	25.8%	Manufacturing scalability requirements	Process standardization	[291]
Biosimilars	USD 25 billion	USD 85 billion	18.7%	Patent expirations, healthcare cost pressures	Competitive manufacturing costs	[292]

## Data Availability

Not applicable.
